# Alteration of *PPAR*‐*GAMMA* (*PPARG*; *PPARγ*) and *PTEN* gene expression in acute myeloid leukemia patients and the promising anticancer effects of *PPARγ* stimulation using pioglitazone on AML cells

**DOI:** 10.1002/mgg3.1818

**Published:** 2021-09-22

**Authors:** Shadi Esmaeili, Sina Salari, Vahid Kaveh, Seyed H. Ghaffari, Davood Bashash

**Affiliations:** ^1^ Department of Hematology and Blood Banking School of Allied Medical Sciences Shahid Beheshti University of Medical Sciences Tehran Iran; ^2^ Department of Medical Oncology, Hematology and Bone Marrow Transplantation Taleghani Hospital Shahid Beheshti University of Medical Sciences Tehran Iran; ^3^ Department of Medical Oncology and Hematology Iran University of Medical Sciences Tehran Iran; ^4^ Hematology, Oncology and Stem Cell Transplantation Research Center Shariati Hospital School of Medicine Tehran University of Medical Sciences Tehran Iran

**Keywords:** acute myeloid leukemia, peroxisome proliferator‐activated receptor‐γ, pioglitazone, *PPARγ*, *PTEN*

## Abstract

**Background:**

In the new era of tailored cancer treatment strategies, finding a molecule to regulate a wide range of intracellular functions is valuable. The unique property of nuclear receptor peroxisome proliferator‐activated receptor‐γ (*PPARγ*; *PPARG*) in transmitting the anti‐survival signals of the chemotherapeutic drugs has fired the enthusiasm into the application of this receptor in cancer treatment.

**Objectives:**

We aimed to investigate the expression of *PPARγ* and one of its downstream targets *PTEN* in non‐M3 acute myeloid leukemia (AML) patients. We also investigated the therapeutic value of *PPARγ* stimulation using pioglitazone in the AML‐derived U937 cell line.

**Methods:**

The blood samples from 30 patients diagnosed with non‐M3 AML as well as 10 healthy individuals were collected and the mRNA expression levels of *PPARγ* and *PTEN* were evaluated. Additionally, we used trypan blue assay, MTT assay, and flow cytometry analysis to evaluate the anti‐leukemic effects of pioglitazone on U937 cells.

**Results:**

While *PTEN* was significantly downregulated in AML patients as compared to the control group, the expression of *PPARγ* was increased in the patients’ group. The expression level of *PPARγ* was also negatively correlated with *PTEN*; however, it was not statistically significant. Besides, *PPARγ* stimulation using pioglitazone reduced survival and proliferative capacity of U937 cells through inducing apoptosis and suppression of cell transition from the G1 phase of the cell cycle.

**Conclusion:**

The results of the present study shed more light on the importance of *PPARγ* and its stimulation in the therapeutic strategies of AML.

## Graphical abstract

The unique property of nuclear receptor peroxisome proliferator‐activated receptor‐γ (PPARγ) in transmitting the anti‐survival signals of the chemotherapeutic drugs has fired the enthusiasm into the application of this receptor in cancer treatment. We found that the expression of PPARγ was increased in non‐M3 AML patients and activation of this receptor using pioglitazone reduced AML cell survival; shedding light on the importance of PPARγ and its ligands in the therapeutic strategies of AML.

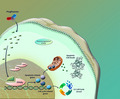

## 1 INTRODUCTION

Conducive molecular targets have become incumbent for the development of new agents for the treatment of human cancers with lower side effects as well as higher anticancer properties. Members of the nuclear receptor superfamily (steroid receptors and their relatives) are putative cancer therapy targets because they function as transcription factors that control the expression of many genes related to the process of carcinogenesis (Safe et al., 2014). Peroxisome proliferator‐activated receptor‐γ (*PPARγ*; *PPARG*; OMIM number *601487) codes for a singular nuclear hormone receptor in the superfamily of ligand‐activated transcriptional factors that is believed to be involved in the regulation of lipid metabolism and has a significant role in the regulation of a broad range of cellular functions, including insulin sensitization, inflammatory responses, and apoptosis (Robbins & Nie, 2012). Aside from these, *PPARγ* has a hand in the propagation of the signaling, mainly through interacting with the well‐known tumor suppressor protein PTEN (translated from the *PTEN* gene; OMIM number *601728) that counteract tumor cells and reduce their survival (Patel et al., 2001). In a considerable number of studies, it has been claimed that the over expression of *PPARγ* in cancer cells is a determining factor in the favorable response rate of neoplastic cells to the anticancer drugs (Papadaki et al., 2005; Sato et al., 2000). The importance of *PPARγ* expression in the therapeutic approaches is to the extent that it has been revealed that the expression of this nuclear receptor is upregulated in cancer cells in the presence of anticancer agents, suggestive of the adjunctive effect of this receptor in the therapeutic strategies (Reddy et al., 2008). This unique characteristic fired an enthusiasm to evaluate whether the stimulation of *PPARγ* in cancer cells could be a promising approach in cancer treatment strategies. From that moment, a stream of PPARγ ligands found their way into cancer research studies to find the best stimulator with the highest anticancer property (Cariou et al., 2012). However, among a long list of natural or synthetic ligands, pioglitazone as an FDA‐approved thiazolidinedione (TZD) drug for the treatment of diabetes type II attracted tremendous attention due to its promising results in preclinical studies (Blanquicett et al., 2008).

In the shadow of controlling the blood glucose level, pioglitazone has shown a valuable efficacy in the reduction of cell survival in different types of human cancers. It has been indicated that pioglitazone could effectively suppress the proliferation and induce apoptosis in breast and prostate cancer cells with elevated expression of *PPARγ* (Fenner & Elstner, 2005; Suzuki et al., 2016). Moreover, the stimulation of *PPARγ* in cancer cells using pioglitazone could induce apoptosis and cell cycle arrest through suppressing the activation of signal transducers and activator of transcription 3 (STAT3) and *BIRC5* (*SURVIVIN*; OMIM number *603352) expression, and enhancing the apoptosis‐inducing factor (AIF) levels in the cells (Tsubaki et al., 2018). Pioglitazone also showed to be a promising agent in the treatment strategy of other solid tumors, including colorectal cancer (Lin et al., 2007), Barrett's carcinoma (Al‐Taie et al., 2009), bladder cancer (Lv et al., 2019), and glioblastoma (Ching et al., 2015). The favorable anticancer effects of pioglitazone have also been reported in acute promyelocytic leukemia (APL), either as a single agent or in combination with chemotherapeutic agents (Esmaeili et al., 2020a, 2020b). Given the promising results of pioglitazone in human leukemia, it was of particular interest to evaluate whether there is a difference in the expression level of *PPARγ* in acute myeloid leukemia (AML) patients and also to examine the therapeutic efficacy of pioglitazone in AML‐derived U937 cells.

## 2 PATIENTS AND METHODS

### 2.1 Sample collection, cell culture, and reagents

For evaluating the expression of *PPARγ* and its downstream target *PTEN* in acute leukemia, the peripheral blood samples from patients diagnosed with de‐novo acute myeloid leukemia (AML) according to French‐American‐British (FAB) classification from October 2018 to August 2019 were collected. Given the distinguished pathogenesis of AML‐M3 patients with other subtypes of AMLs, the samples from this group (13.5% of total samples) were excluded from our study. Table 1 provided the characteristics of 30 non‐M3 AML patients. Among all patients, 56% (17 out of 30) were male and 44% (13 out of 30) were female with an average age of 49 years old for both groups. To better analyze our data, we also collected the blood from 10 healthy counterparts. The utilized research protocol in the present study was approved by the Research Ethics Committee at the Shahid Beheshti University of Medical Sciences (IR.SBMU.RETECH.REC.1399.310). The informed consent according to the statement of Helsinki was also given to all participants.

**TABLE 1:** Nucleotide sequences of primers used for real‐time RT‐PCR

Gene	Forward primer (5′−3′)	Reverse primer (5′−3′)	Size (bp)
*ABL1*	CTTCTTGGTGCGTGAGAGTGAG	GACGTAGAGCTTGCCATCAGAAG	115
*PTEN*	CACACGACGGGAAGACAAGTTC	TCCTCTGGTCCTGGTATGAAGAATG	162
*PPARγ*	GGGATCAGCTCCGTGGATCT	TGCACTTTGGTACTCTTGAAGTT	186

Abbreviations: *ABL1*, NM_007313.3; *PPARγ*: NM_001354666.3; *PTEN*, NM_000314.8.

### 2.2 Sample preparation and gene expression analysis

Once after the blood collection, peripheral blood mononuclear cells (PBMCs) were isolated by Ficoll‐Hypaque (Lymphodex, inno‐Train) density gradient centrifugation at 400g for 20 min. The isolated PBMCs were washed twice with PBS and then their RNAs were extracted with the High Pure RNA isolation kit as recommended by the provider (Qiagen). After confirming the quantity of the extracted RNA by Nanodrop ND‐1000 (Optical Density (OD) 260/280 nm ratio >1.8), the reverse transcription reaction was performed using the cDNA synthesis Thermo fermentas kit (Thermoscientific). For gene expression analysis, the relevant synthesized cDNAs were subjected to quantitative real‐time PCR (qRT‐PCR) using SYBR Premix Ex Taq technology (Takara BIO) on a light cycler instrument (Roche Diagnostics). We provided a reaction mixture with the final volume of 15 μl containing 2 μl cDNA, 1 μl of forward and reverse primers (10 pmol), 7.5 μl of Master Mix, and 4.5 μl of water. The reaction mixture was then placed in a light cycler instrument with the thermal cycling schedule explained in our previous studies (Safaroghli‐Azar et al., 2019). Phosphoribosyl *ABL1* (OMIM number *189980; NM_007313.3) was amplified as the housekeeping gene and fold change in expression of the target genes *PPARG* (NM_001354666.3) and *PTEN* (NM_000314.8) relative to *ABL1* was calculated on the basis of comparative on 2^−ΔΔCt^ relative expression formula. The sequences of the primers used for Real‐Time RT‐PCR are listed in Table 1.

### 2.3 Cell culture and drug treatment

The human cell line of AML U937 was grown in RPMI 1640 medium supplemented with 10% fetal bovine serum (Gibco) and 1% penicillin/streptomycin maintained in 5% CO_2_ at 37°C in a humidified incubator. After reaching the proper confluency, U937 cells were treated with increasing concentrations of PPARγ ligand, pioglitazone, which its stock was provided by resolving the powder in sterile dimethyl sulfoxide (DMSO). In addition to the negative control (no inhibitor), U937 cells were treated with the corresponding concentration of DMSO as an alternative negative control.

### 2.4 Trypan blue exclusion test of cell count and viability

The trypan blue assay was used to evaluate the viability of U937 cells upon treatment with increasing concentrations of pioglitazone. After drug treatment and at the relevant time interval, 20 µl from each sample was collected and were mixed with 20 µl of 0.4% trypan blue (Invitrogen). The mixtures were incubated at room temperature for 1–2 min and then were loaded onto a Neubauer hemocytometer for manually calculating cell viability.

### 2.5 MTT assay

U937 cells (5 × 10^3^ cells) were cultured in a 96‐well plate and were treated with increasing concentrations of pioglitazone (0–250 µM). Plates were incubated in a humidified incubator at 37°C up to 48 h. After each time interval, 100 μl of MTT solution (5 mg/ml in PBS) was added to each well, and plates were incubated at 37°C for a further 3 h. Then, the plates were centrifuged, the media was discarded and 100 μl of DMSO was added to each well to resolve the formazan crystals. The absorbance of each well was measured at 570 nm in an ELISA reader.

### 2.6 Cell cycle distribution analysis

To investigate the effect of pioglitazone on cell cycle progression, propidium iodide (PI) staining was used. After treating 10^6^ U937 cells with pioglitazone for 24 h, the cells were centrifuged and the cell pellets were first washed twice with cold PBS and then fixed with 70% ethanol. Prior to the staining with 50 μg/ml PI stain, the fixed cells were treated for half an hour with 0.5 μg/ml RNase in PBS at 37°C. Flow cytometry (Partec PASIII flow cytometry) and Windows FlowJo V10 software were used to analyze the cellular DNA content.

### 2.7 Assessment of apoptosis using flow cytometry

The annexin‐V/PI staining assay was used to evaluate the externalization of phosphatidylserine (PS) upon treatment of the cells with pioglitazone. A similar procedure to cell cycle distribution analysis was done with the difference that the harvested cells after 24 h of drug treatment were suspended in 100 µl of the incubation buffer. Then, cells were mixed with annexin‐V Flous (2 μl/sample) for 20 min in the dark and the emitted fluorescence was measured using flowcytometery (Partec PASIII flow cytometry). The percentage of annexin‐V and annexin‐V/PI double‐positive cells was calculated using Windows FlowJo V10 software.

### 2.8 Statistical analysis

All the statistical analyses, either for patients’ samples or cell line was conducted using SPSS software (version16.0) and the GraphPad Prism6 software. The independent Student's *t*‐test was used for comparing the results obtained from patients or cell lines with the relevant control groups. The Mann–Whitney *U*‐test was used to compare gene expression between the patient and control groups as a nonparametric test of the null hypothesis. All experiments were done in duplicate or triplicate. A probability level of *p* ≤ 0.05 was considered statistically significant.

## 3 RESULTS

### 3.1 Patient characteristics

Blood samples of 30 patients diagnosed with non‐M3 AML with a blast percentage of more than 20%, including 17 males and 13 females were collected. The median age was 49, ranging from 19 to 83 years. We also collected the blood from 10 healthy individuals with a median age close to the patients’ group. The characteristics of AML patients, including FAB subtype, Hb, HCT, number of RBCs, WBCs, platelets, and also blast percentage are summarized in Table 2.

**TABLE 2:** The clinical characteristics of de‐novo non‐M3 acute myeloid leukemia patients

No	FAB	RBC × 10^6^	WBC × 10^3^	PLT × 10^3^	Hb (g/dl)	HCT (%)	Blast (%)
1	AML‐M2	3.32	24.7	48	9.7	28.4	35
2	AML‐M1	2.79	50.01	165	9.2	27.9	50
3	AML‐M2	3.12	86.45	78	9.5	28.1	65
4	AML‐M2	4.41	32.14	170	12.9	38.7	40
5	AML‐M0	2.98	45.24	93	10.1	28. 8	35
6	AML‐M4	2.71	23.39	238	8.7	26.1	25
7	AML‐M2	3.58	14.46	24	10.5	29.3	60
8	AML‐M5	3.5	37.06	55	9.8	31.6	25
9	AML‐M2	2.9	46.1	38	9.3	28.3	45
10	AML‐M4	3.53	11.11	80	10.6	31.3	80
11	AML‐M2	5.34	15.34	160	14.6	42.1	45
12	AML‐M1	4.9	37.2	121	10	32	55
13	AML‐M1	2.31	108	10	6.01	21	40
14	AML‐M2	4.1	36.45	78	11.39	34	80
15	AML‐M4	5.01	13	175	14.6	42.14	80
16	AML‐M5	3.03	36.54	45	9.5	27.3	90
17	AML‐M4	3.9	29.3	98	9.1	29	43
18	AML‐M4	4.18	16.36	189	12.3	38.05	26
19	AML‐M6	4.45	17.45	173	13.5	40.4	32
20	AML‐M2	3.56	54.04	121	11.6	34.4	41
21	AML‐M1	3.06	14.4	63	9.3	26.6	21
22	AML‐M2	2.75	29.5	69	10.3	31.4	38
23	AML‐M4	3.43	27.76	91	9.5	31.3	41
24	AML‐M2	3.37	106.7	35	9.4	29.2	68
25	AML‐M1	3.7	23	96	10.1	29.4	51
26	AML‐M2	3.33	16.86	31	10.3	30.2	65
27	AML‐M4	4.17	18.54	26	9.8	28.1	35
28	AML‐M5	3.67	21.41	32	8.6	26.1	40
29	AML‐M2	4.02	13.52	15	11.7	34	30
30	AML‐M1	2.89	42.51	62	9.9	29.41	28

Abbreviations: Hb, hemoglobin; PLT, platelet; RBC, red blood cells; WBC, white blood cells.

### 3.2 *PPARγ* and *PTEN* mRNA expression levels in AML patients and healthy counterparts

**FIGURE 1:**
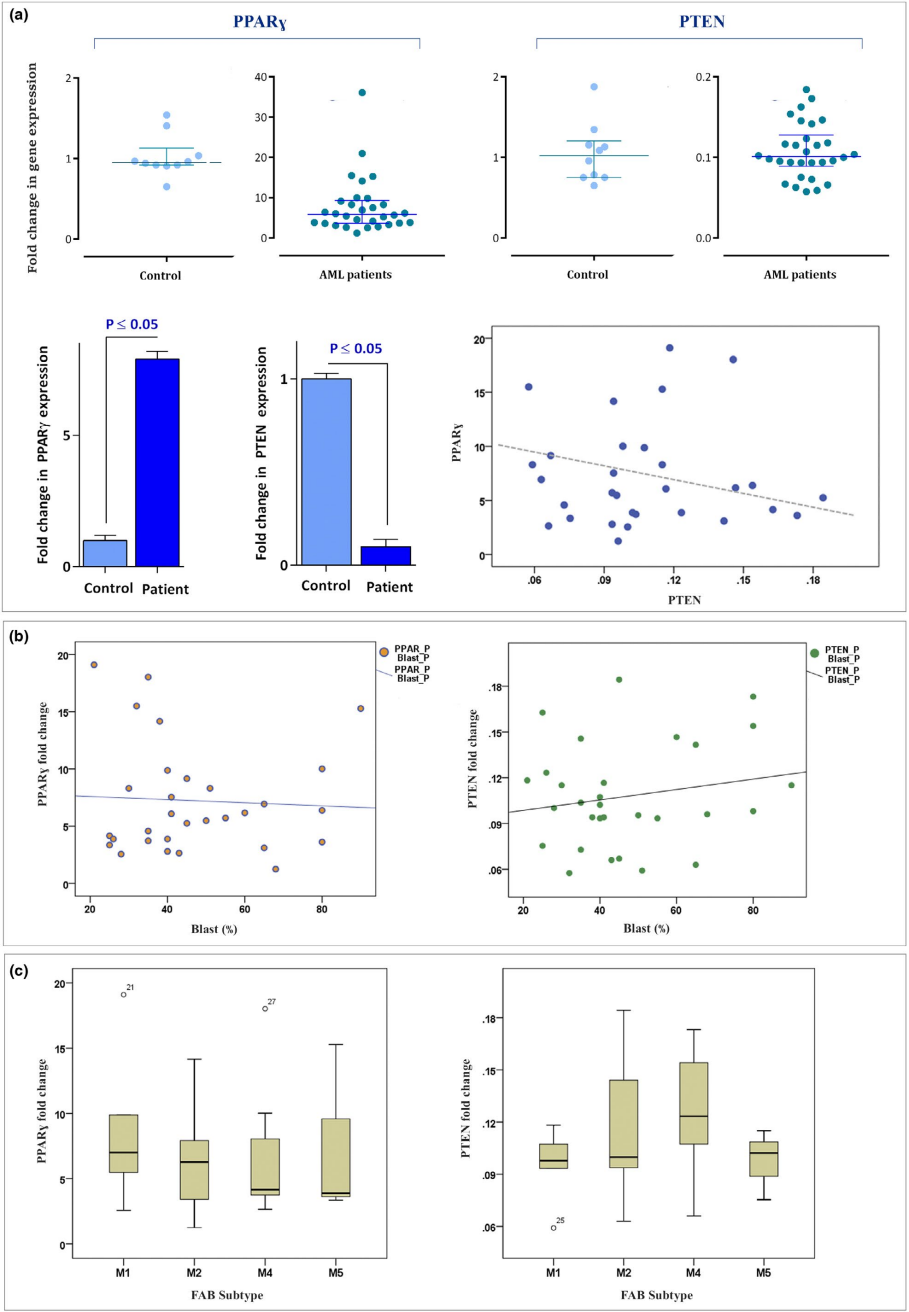
The mRNA expression levels of *PPARγ* and *PTEN* in newly diagnosed non‐M3 AML patients. (a) While the expression levels of *PPARγ* (NM_001354666.3) in AML patients were significantly higher than the control group, the mRNA level of *PTEN* (NM_000314.8) was significantly decreased. Values are given as mean ± SD of three independent experiments. The expression level of *PPARγ* was also negatively correlated with *PTEN*; however, it was not statistically significant (*r* = −0.35, *p* < 0.85). (b) We could not find any significant correlation between blast percentage with either *PPARγ* (*r* = 0.002, *p* < 0.99) or *PTEN* expressions (*r* = 0.048, *p* < 0.8). (c) The results of *PPARγ* and *PTEN* gene expression according to the FAB subtype were represented. As shown, *PPARγ* expression was higher in patients with dominant myeloblasts (M1 and M2 subtypes) compared to those with myelomonocytic cells (M4‐M5); on the other hand, AML cases with involvement of myelomonoblastic cells (M4) exhibited a higher level of *PTEN* mRNA

Given the tight association with the well‐known tumor suppressor protein PTEN, *PPARγ* and its associated signaling found value in the pathogenesis of different human cancers (Patel et al., 2001). Although the positive correlation between the expression of *PTEN* and *PPARγ* was reported in most cases of solid tumors (Teresi et al., 2006), the expression of these genes and their association with leukemogenesis has not yet been clarified. The results of gene expression analysis in 30 collected blood from AML diagnosed patients and 10 healthy individuals revealed that unlike the expression of *PTEN* which significantly displayed a decreased expression in the patients’ group as compared to the control group, the expression of *PPARγ* had an upward trend in AML patients. The expression level of *PPARγ* was also negatively correlated with *PTEN*; however, it was not statistically significant (Pearson's correlation; *r* = −0.35, *p* < 0.85). As the percentage of blasts was widely different from one patient to another, we also evaluated the correlation between blast percentage and *PPARγ* and *PTEN* expression. As represented in Figure 1b, we could not find any significant correlation between blast percentage with neither *PPARγ* (*r* = 0.002, *p* < 0.99) nor *PTEN* expressions (*r* = 0.048, *p* < 0.8). The results of *PPARγ* and *PTEN* gene expression according to the FAB subtype were also represented in Figure 1c. As shown, *PPARγ* expression was higher in patients with dominant myeloblasts (M1 and M2 subtypes) compared to those with myelomonocytic cells (M4‐M5); on the other hand, AML cases with involvement of myelomonoblastic cells (M4) exhibited a higher level of *PTEN* mRNA. Notably, we analyzed neither M0 nor M6 as there was only one case of each subtype.

### 3.3 Pioglitazone inhibited U937 cell growth and viability

**FIGURE 2:**
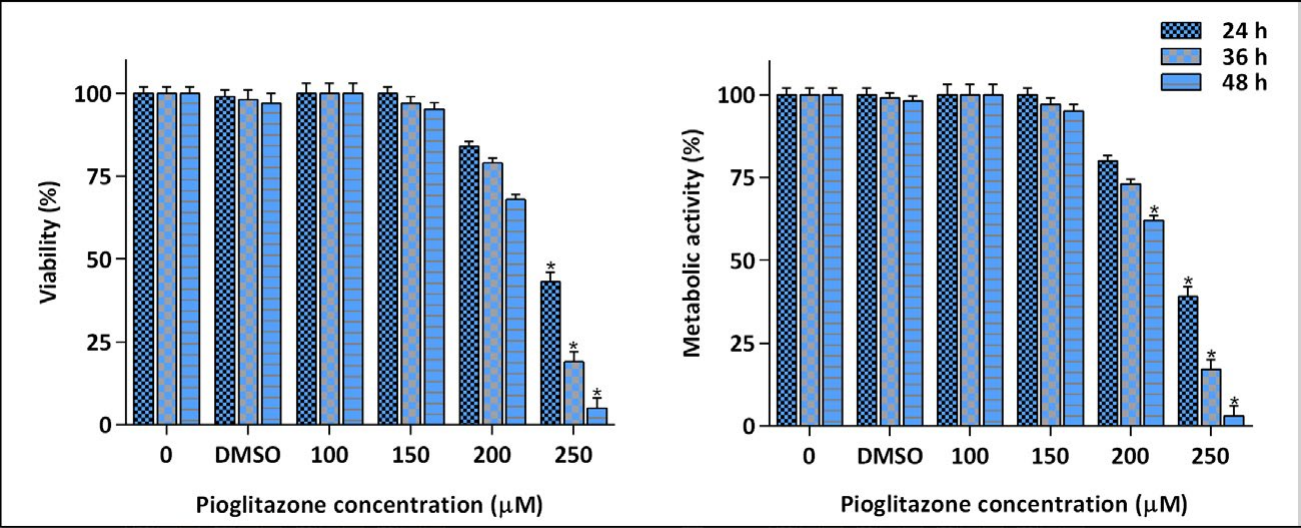
The antileukemic effect of pioglitazone on U937 cells. The results obtained from trypan blue and MTT assays revealed that pioglitazone could diminish the proliferative and survival capacity of U937 cells. Values are given as mean ± SD of three independent experiments. *p ≤ 0.05 represented significant changes from the control

The upregulation in the expression level of *PPARγ* in AML patients raised the question that how the expression of a tumor suppressor protein was increased in malignant cases. We hypothesis that probably in the absence of PPARγ‐ associated ligand, the expression of this receptor compensatory increased in leukemic cells, which could be then recruited for the treatment strategies. Given this, we decided to evaluate whether the stimulation of *PPARγ* in leukemic cells is associated with the reduction of cell viability. We treated AML‐derived U937 cells with increasing concentrations of pioglitazone, one of the most important ligands of PPARγ, and then the viability and the proliferative capacity of the cells were evaluated using trypan blue and MTT assays. Our results showed that pioglitazone significantly diminished the viability of U937 cells in both time‐ and concentrations‐dependent manner (Figure 2). In agreement, the results of the MTT assay also showed that in the presence of pioglitazone, there was a significant reduction in the metabolic activity of U937 cells. As presented in Figure 2, after the treatment of U937 cells with pioglitazone at the concentration of 250 µM for 48 h, only 3% of the cells found the chance to maintain their metabolic activity. Taken together, these findings shed light on the value of PPARγ and its ligand pioglitazone in counteracting with the ability of leukemic cells to sustain their survival and proliferative capacity.

### 3.4 The effect of pioglitazone on the distribution of U937 in different stages of the cell cycle

**FIGURE 3:**
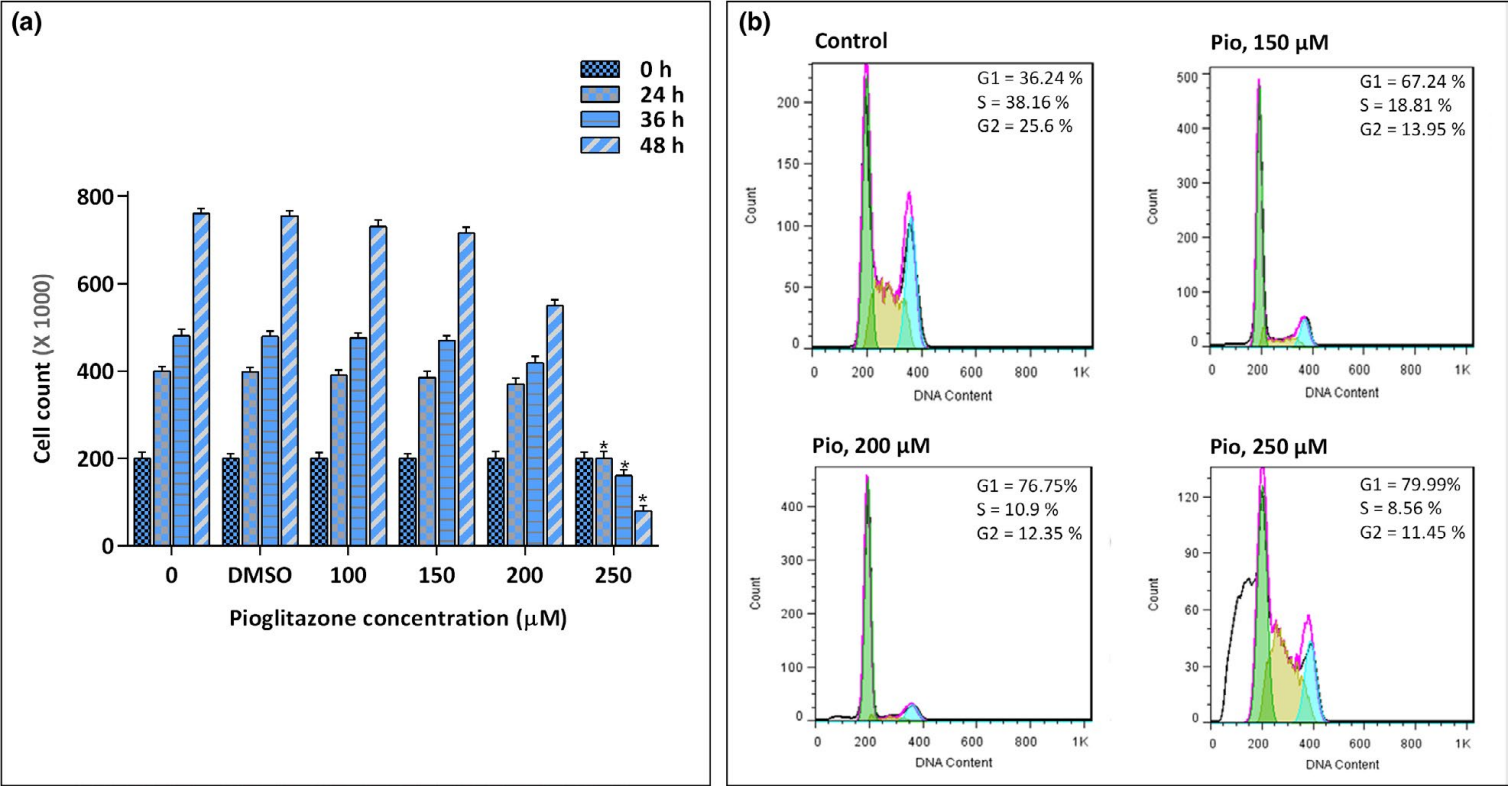
The effect of pioglitazone on the distribution of U937 cells in different phases of the cell cycle. (a) Upon treatment of U937 cells with pioglitazone, there was a significant reduction in the number of viable cells. (b) The antiproliferative effect of pioglitazone on U937 cells was mediated through induction of G1 cell cycle arrest. Values are given as mean ± SD of three independent experiments. *p ≤ 0.05 represented significant changes from the control

Evaluating the effect of pioglitazone on the growth kinetics of U937 cells revealed that the stimulation of *PPARγ* in the leukemic cells was coupled with the remarkable reduction in the number of cells (Figure 3a). This finding encouraged us to evaluate whether the antiproliferative effect of pioglitazone of leukemic cells is associated with the alteration in the distribution of the cells in different phases of the cell cycle. The PI staining assay showed that pioglitazone decreased the percentage of cells in S and G2/M phases from 38.16% and 25.6% in the control group to 8.56% and 11.45% in 250 µM‐treated cells, respectively (Figure 3b). In addition, treatment with pioglitazone resulted in a significant accumulation of the cells in the G1 phase (Figure 3b); indicating that the growth‐suppressive effect of the inhibitor is mediated, at least partly, through induction of G1 arrest.

### 3.5 Stimulation of *PPARγ* in U937 cells using pioglitazone was coupled with the induction of apoptosis

**FIGURE 4:**
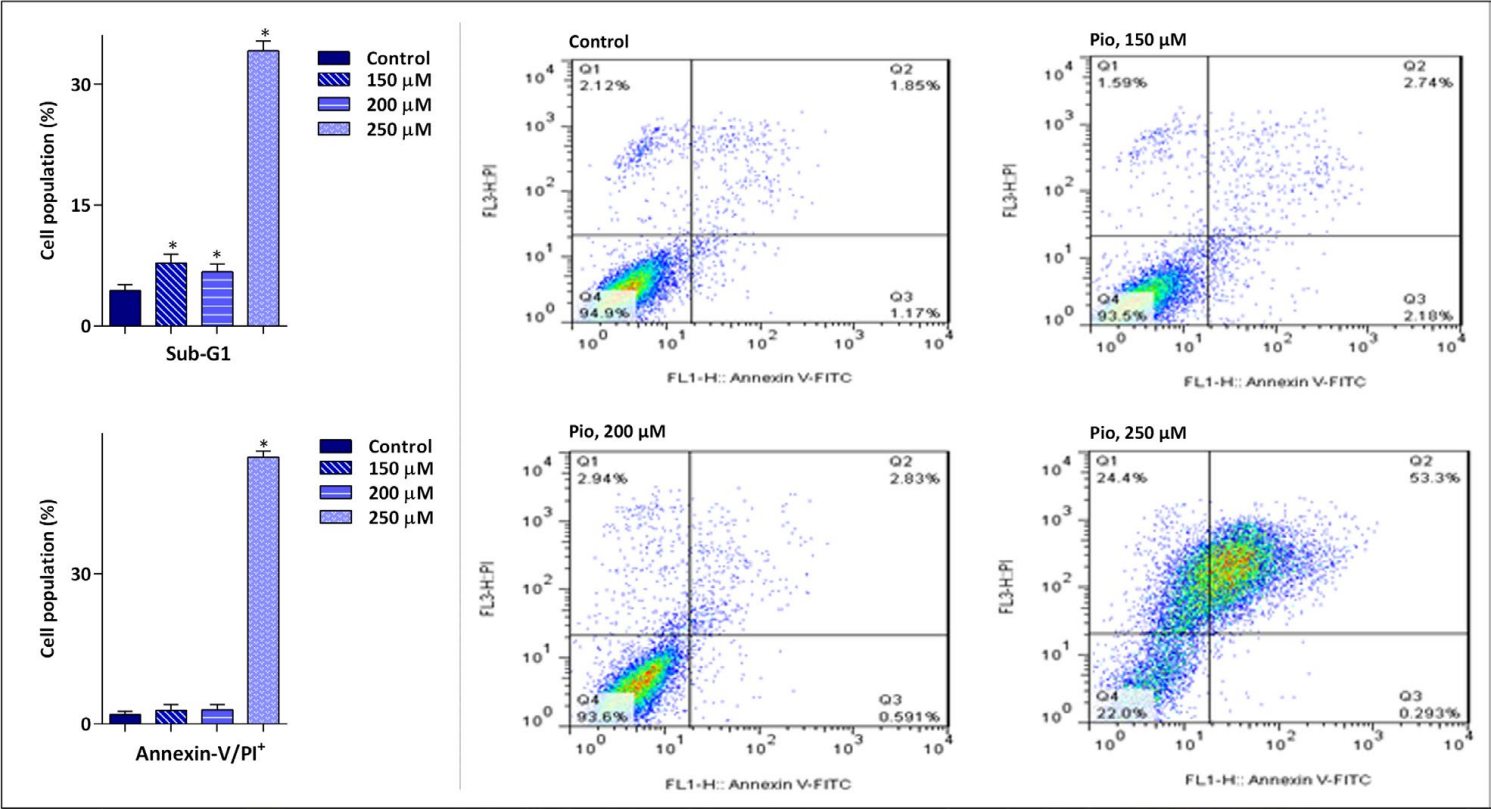
The activation of *PPARγ* in U937 was coupled with the induction of apoptosis. Measuring the effect of pioglitazone on the proportion of the cells in sub‐G1 revealed that *PPARγ* stimulation in U937 cells elevated the fraction of hypodiploid cells. Moreover, we found that the number of Annexin‐V/PI double‐positive inhibitor‐treated cells was increased in response to drug treatment in U937 cells, as compared with the untreated group. Values are given as mean ± SD of three independent experiments. *p ≤ 0.05 represented significant changes from the control

As a noble member of nuclear receptors, the regulatory role of *PPARγ* in the induction of apoptotic cell death has been reported in a mounting body of evidence (Elrod & Sun, 2008; Zhang et al., 2007). Our results also showed that stimulation of this receptor in U937 cells using pioglitazone could arrest the cell transition from the sub‐G1 phase of the cell cycle. As depicted in Figure 4, the percentage of the cells settled in the sub‐G1 phase was increased from 4.4% in the control group to 34.13% in the 250 µM‐treated group. To confirm our results, we evaluated the externalization of phosphatidylserine (PS) on the surface of U937 cells using flow cytometry. In corroboration with the elevated cell population in sub‐G1, we found that treatment of cells with pioglitazone resulted in an increased percentage of Annexin‐V/PI double‐positive cells as compared to the control group (Figure 4), suggestive of the apoptotic effect of the agent on leukemic cells.

## 4 DISCUSSION

Acute myeloid leukemia (AML) is one of the most heterogeneous malignancies that day by day more molecules are identified to participate in its pathogenesis. Despite the significant advances in the identification of new targets in the treatment of AML, still, no appropriate protocol has been developed for patients, especially for those with advanced age, who mostly receive supportive treatment rather than concrete anticancer‐based therapies (Eleni et al., 2010; Sekeres & Stone, 2002). Undeniably, the toxicity and the unfavorable adverse effects of chemotherapeutic agents that are intolerable for the majority of elder patients are the main reason why the supportive tactics are better rather than the cytotoxic strategy (Eleni et al., 2010). Given this major obstacle, efforts have now been made to identify an agent that not only can inhibit the survival of cancer, but also has the least adverse effects on patients. Pioglitazone, a valuable member of thiazolidinediones, is a drug that is originally used for the treatment of type II diabetes (DeFronzo et al., 2019). The results of the previous investigations declared that pioglitazone at concentrations between 15 and 45 mg/day could reduce the blood glycosylated hemoglobin (HbA1c) levels in patients with type 2 diabetes mellitus (Gillies & Dunn, 2000).

Based on its regulatory impact on glucose metabolism, which is highly activated in cancer cells, low adverse effects, and high tolerability rate, it seems that pioglitazone could be a promising candidate for incorporation into the treatment protocol of human cancers (Blanquicett et al., 2008). The results of the molecular investigations declared that in addition to the suppressive effect of the agent on metabolic activities, the significance of pioglitazone in cancer treatment is due to its ability to interact with nuclear receptor peroxisome proliferator‐activated receptor‐γ (PPARγ; Ninomiya et al., 2014; Saiki et al., 2006), which is one of the most important receptors for transmitting the anti‐survival signals in the malignant cells (Shen et al., 2012). Another unique property of pioglitazone, which guarantees its low side effects as well as its vigorous anticancer effects, is its selective behavior on cells with overexpressed *PPARγ*. Saiki et al. reported that as compared to the cells with normal or lower expression of *PPARγ*, pioglitazone at the concentration ranging from 100 to 300 µM induced significant anti‐survival and antiproliferative effects on *PPARγ*‐expressing cancer cells (Saiki et al., 2006). In agreement, Hata et al. also designed a study to evaluate the effect of pioglitazone on different leukemic cell lines (K562, HL60, U937, HEL, CEM, and NALM1). Their results indicated that pioglitazone up to the concertation of 300 µM did not exert any cytotoxic effects on normal cells (Hatta et al., 2004). Moreover, the results of several clinical trials on both AML and CML patients indicated that pioglitazone at the dose of 45 mg/day is safe and well‐tolerated (Ghadiany et al., 2019; Rousselot et al., 2017). Prost et al. delineated that when imatinib‐resistant CML patients were treated with pioglitazone, the overall survival increased up to 4.7 years. Given this, they extended their study to CD34^+^ CML cells and concluded that pioglitazone may have the ability to eradicate the population of leukemic stem cells (LSCs)—a group of neoplastic cells which widely participate in induction of chemo‐resistance (Prost et al., 2015). Ghadiany et al. have also administrated pioglitazone together with cytarabine and daunorubicin to newly diagnosed AML patients and indicated that combination could increase the survival of the patients (Ghadiany et al., 2019). Based on this finding, we first evaluated the expression level of this nuclear receptor in the PBMNCs of patients with AML.

The result of our experiments was suggestive of the significant elevation in the mRNA expression level of *PPARγ* in non‐M3 AML patients at diagnosis as compared with the healthy counterparts; suggesting that probably the activation of *PPARγ* in leukemic cells could act against the survival of cancer cells. This finding was in accordance with the previous studies reporting the upregulation of *PPARγ* in human pancreatic carcinoma (Sun et al., 2009), colorectal cancer (Lee et al., 2006), and breast cancer (Sporn et al., 2001). As the most important partner of *PPARγ*, numerous studies have declared that the tumor‐suppressive effects of PPARγ are exerted through upregulation of PTEN, which gained its reputation due to the involvement in the PI3K signaling axis (Farrow & Evers, 2003; Patel et al., 2001). However, in some cases, PPARγ could induce cytotoxic effects independent of this tumor suppressor (Jagan et al., 2013). Kenneth et al. have indicated that pioglitazone could halt the proliferation of *PTEN*‐deficient lung cancer cells and enhance their sensitivity to EGFR tyrosine kinase inhibitor (To et al., 2018); shedding light on the ability of pioglitazone to induce cell death in cancer cells with mutant *PTEN*. Unlike upregulation in *PPARγ* expression, we found that the mRNA expression level of *PTEN* had a noteworthy reduction in patients as compared to the control group. Indeed, it became evident that *PPARγ* expression was in a negative association with *PTEN* expression; while *PTEN* was significantly downregulated in AML patients, the expression of *PPARγ* was increased in AML cases as compared to the control group.

The overexpression of *PPARγ* in AML cells shed light on the likelihood of pioglitazone efficacy in AML‐derived U937 cells. Interestingly, our results showed that the activation of *PPARγ* in U937 cells using pioglitazone was coupled with the reduction in the viability and growth of leukemic cells, as revealed by the significant reduction in the number of cells and the metabolic activity. Saiki et al. indicated that pioglitazone could reduce the colony‐forming ability of U937 cells in the presence of growth factors such as CFU‐GM and CFU‐E. Moreover, they delineated that when this agonist stimulates *PPARγ*, it could induce G1 cell cycle arrest in APL‐derived HL60 cell line (Saiki et al., 2006). Consistently, Lee et al. (2006) demonstrated that pioglitazone inhibited cell growth and induced apoptosis in Rb‐deficient human colorectal cancer cells. In another study, it has also been claimed that pioglitazone could reduce the survival of acute promyelocytic leukemia (APL) cells by elevating the intracellular level of reactive oxygen species (ROS) either as a single agent or in combination with arsenic trioxide (Esmaeili et al., 2020b).

**FIGURE 5:**
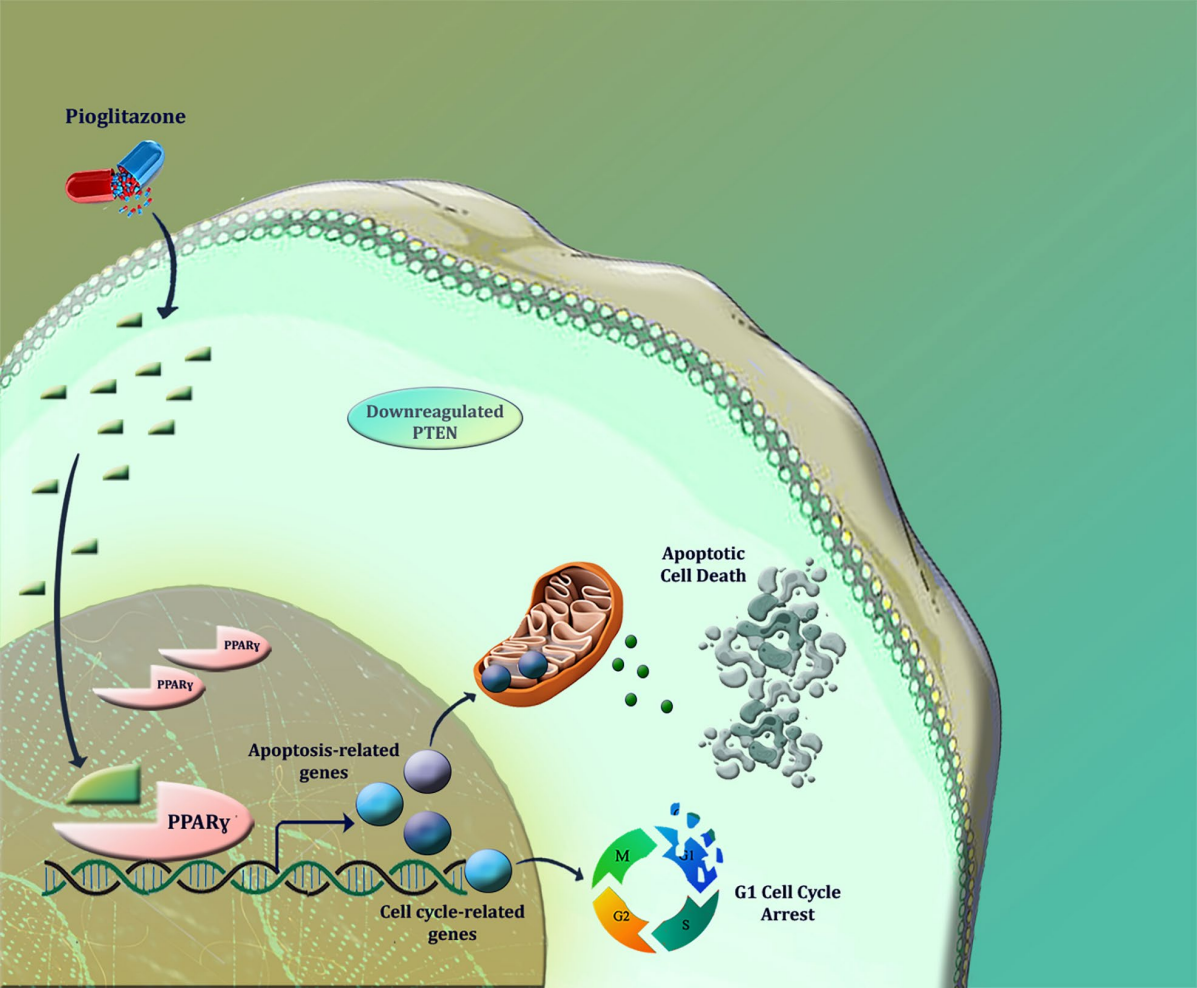
A schematic representation proposed for the promising anticancer effects of *PPARγ* stimulation using pioglitazone on AML cells. The results of this study revealed that *PPARγ* upregulation were coupled with downregulated *PTEN* in AML patients as compared to the control group. As represented, stimulation of *PPARγ* using pioglitazone reduced the AML‐derived U937 cell survival, at least partly, through inducing apoptosis and suppression of cell transition from the G1 phase of the cell cycle

Previous studies indicated that PPARγ and its associated signaling pathway have a deep role in the regulation of cell proliferation through controlling the expression of the genes participating in the progression of the cell cycle (Müller et al., 2008). Moreover, a mounting body of studies has suggested that the activation of PPARγ in cancer cells could induce apoptotic signals by altering the balance between the expression level of pro‐ and anti‐apoptotic target genes (Elrod & Sun, 2008). Of great interest, when we stimulated *PPARγ* in U937 cells, we found not only the transition of the leukemic cells from the G1 phase of the cell cycle was blocked, but also there was a significant increase in the number of the cells that underwent apoptotic death; suggestive of the efficacy of pioglitazone in the treatment of mutant *PTEN*‐expressing AML‐derived U937 cells. Notably, the results of our previous study revealed that while pioglitazone could inhibit the viability of wild‐type *PTEN*‐expressing NB4, it failed to induce significant cytotoxicity both in KG1 cells with wild‐type *PTEN* and K562 cells harboring mutant *PTEN* (Esmaeili et al., 2020b); further indicating that other factors such as basal expression of *PPARγ* as well as different molecular characteristics of leukemic cells may affect the ability of pioglitazone to induce apoptosis in leukemic cells. To provide a better prospect, we designed a schematic figure to represent a summary of our findings (Figure 5). Taken together, the results obtained from this study shed light on the importance of *PPARG* in the treatment strategies of AML and suggested that the stimulation of this nuclear receptor using pioglitazone may provide a promising outcome for patients suffering from non‐M3 AML.

## CONFLICT OF INTEREST

The authors declare that they have no conflict of interest.

## AUTHOR CONTRIBUTIONS

Shadi Esmaeili: Acquisition of data, analysis and interpretation of data, writing‐original draft preparation, reviewing, and editing. Sina Salary: Acquisition of data, technical help, writing‐reviewing. Vahid Kaveh: Acquisition of data, technical help, and writing‐reviewing. S.H. Ghaffari: Conceptualization, writing‐reviewing, and editing. Davood Bashash: Conceptualization, analysis and interpretation of data, writing‐reviewing, and editing, approved the published version.

## 5 ETHICS STATEMENT

This study was approved by the Research Ethics Committee at the Shahid Beheshti University of Medical Sciences (IR.SBMU.RETECH.REC.1399.310).

## Data Availability

Data sharing not applicable to this article as no datasets were generated or analyzed during the current study.

## References

[mgg31818-bib-0001] Al‐Taie, O. H. , Graf, T. , Illert, B. , Katzenberger, T. , Mörk, H. , Kraus, M. R. , Barthelmes, H. U. , Scheurlen, M. , & Seufert, J. (2009). Differential effects of PPARγ activation by the oral antidiabetic agent pioglitazone in Barrett’s carcinoma in vitro and in vivo. Journal of Gastroenterology, 44(9), 919–929. 10.1007/s00535-009-0086-y 19506796

[mgg31818-bib-0002] Blanquicett, C. , Roman, J. , & Hart, C. M. (2008). Thiazolidinediones as anti‐cancer agents. Cancer Therapy, 6(A), 25. 19079765 PMC2600565

[mgg31818-bib-0003] Cariou, B. , Charbonnel, B. , & Staels, B. (2012). Thiazolidinediones and PPARγ agonists: Time for a reassessment. Trends in Endocrinology & Metabolism, 23(5), 205–215. 10.1016/j.tem.2012.03.001 22513163

[mgg31818-bib-0004] Ching, J. , Amiridis, S. , Stylli, S. S. , Morokoff, A. P. , O’Brien, T. J. , & Kaye, A. H. (2015). A novel treatment strategy for glioblastoma multiforme and glioma associated seizures: Increasing glutamate uptake with PPARγ agonists. Journal of Clinical Neuroscience, 22(1), 21–28. 10.1016/j.jocn.2014.09.001 25439749

[mgg31818-bib-0005] DeFronzo, R. A. , Inzucchi, S. , Abdul‐Ghani, M. , & Nissen, S. E. (2019). Pioglitazone: The forgotten, cost‐effective cardioprotective drug for type 2 diabetes. Diabetes and Vascular Disease Research, 16(2), 133–143. 10.1177/1479164118825376 30706731

[mgg31818-bib-0006] Eleni, L. D. , Nicholas, Z. C. , & Alexandros, S. (2010). Challenges in treating older patients with acute myeloid leukemia. Journal of Oncology, 2010, 1–11. 10.1155/2010/943823 PMC2902223 20628485

[mgg31818-bib-0007] Elrod, H. A. , & Sun, S.‐Y. (2008). PPARγ and apoptosis in cancer. PPAR research 2008. 10.1155/2008/704165 PMC2442903 18615184

[mgg31818-bib-0008] Esmaeili, S. , Safaroghli‐Azar, A. , Pourbagheri‐Sigaroodi, A. , Salari, S. , Gharehbaghian, A. , & Bashash, D. (2020a). Activation of PPARγ intensified the effects of arsenic trioxide in acute promyelocytic leukemia through the suppression of PI3K/Akt pathway: Proposing a novel anticancer effect for pioglitazone. The International Journal of Biochemistry & Cell Biology, 122, 105739. 32169580 10.1016/j.biocel.2020.105739

[mgg31818-bib-0009] Esmaeili, S. , Safaroghli‐Azar, A. , Pourbagheri‐Sigaroodi, A. , Salari, S. , Gharehbaghian, A. , Hamidpour, M. , & Bashash, D. (2020b). Stimulation of peroxisome proliferator‐activated receptor‐gamma (PPARγ) using pioglitazone decreases the survival of acute promyelocytic leukemia cells through up‐regulation of PTEN expression. Anti‐Cancer Agents in Medicinal Chemistry. 21(1), 108–119. 10.2174/1871520620666200817101533 32807067

[mgg31818-bib-0011] Farrow, B. , & Evers, B. M. (2003). Activation of PPARγ increases PTEN expression in pancreatic cancer cells. Biochemical and Biophysical Research Communications, 301(1), 50–53. 10.1016/S0006-291X(02)02983-2 12535639

[mgg31818-bib-0012] Fenner, M. H. , & Elstner, E. (2005). Peroxisome proliferator‐activated receptor‐gamma ligands for the treatment of breast cancer. Expert Opinion on Investigational Drugs, 14(6), 557–568. 10.1517/13543784.14.6.557 16004588

[mgg31818-bib-0013] Ghadiany, M. , Tabarraee, M. , Salari, S. , Haghighi, S. , Rezvani, H. , Ghasemi, S. N. , & Karimi‐Sari, H. (2019). Adding oral pioglitazone to standard induction chemotherapy of acute myeloid leukemia: A randomized clinical trial. Clinical Lymphoma Myeloma and Leukemia, 19(4), 206–212. 10.1016/j.clml.2019.01.006 30770307

[mgg31818-bib-0014] Gillies, P. S. , & Dunn, C. J. (2000). Pioglitazone. Drugs, 60(2), 333–343. 10.2165/00003495-200060020-00009 10983737

[mgg31818-bib-0015] Hatta, Y. , Saiki, M. , Enomoto, Y. , Aizawa, S. , Sawada, U. , & Horie, T. (2004). Pioglitazone Inhibits the Growth of Human Leukemic Cell Lines and Primary Leukemic Cells in Vitro. Blood, 104(11), 4493. 10.1182/blood.V104.11.4493.4493 16820887

[mgg31818-bib-0016] Jagan, I. , Fatehullah, A. , Deevi, R. K. , Bingham, V. , & Campbell, F. C. (2013). Rescue of glandular dysmorphogenesis in PTEN‐deficient colorectal cancer epithelium by PPARγ‐targeted therapy. Oncogene, 32(10), 1305–1315. 10.1038/onc.2012.140 22543585 PMC3446865

[mgg31818-bib-0017] Lee, C. J. , Han, J. S. , Seo, C. Y. , Park, T. H. , Kwon, H. C. , Jeong, J. S. , Kim, I. H. , Yun, J. , Bae, Y. S. , Kwak, J. Y. , & Park, J. I. (2006). Pioglitazone, a synthetic ligand for PPARγ, induces apoptosis in RB‐deficient human colorectal cancer cells. Apoptosis, 11(3), 401–411. 10.1007/s10495-006-4003-z 16520894

[mgg31818-bib-0018] Lin, M. S. , Chen, W. C. , Bai, X. , & Wang, Y. D. (2007). Activation of peroxisome proliferator‐activated receptor γ inhibits cell growth via apoptosis and arrest of the cell cycle in human colorectal cancer. Journal of Digestive Diseases, 8(2), 82–88. 17532820 10.1111/j.1443-9573.2007.00290.x

[mgg31818-bib-0019] Lv, S. , Wang, W. , Wang, H. , Zhu, Y. , & Lei, C. (2019). PPARγ activation serves as therapeutic strategy against bladder cancer via inhibiting PI3K‐Akt signaling pathway. BMC Cancer, 19(1), 1–13. 10.1186/s12885-019-5426-6 30845932 PMC6407222

[mgg31818-bib-0020] Müller, R. , Rieck, M. , & Müller‐Brüsselbach, S. (2008). Regulation of cell proliferation and differentiation by PPARβ/δ. PPAR Research, 2008, 1–5. 10.1155/2008/614852 PMC2542843 18815620

[mgg31818-bib-0021] Ninomiya, I. , Yamazaki, K. , Oyama, K. , Hayashi, H. , Tajima, H. , Kitagawa, H. , Fushida, S. , Fujimura, T. , & Ohta, T. (2014). Pioglitazone inhibits the proliferation and metastasis of human pancreatic cancer cells. Oncology Letters, 8(6), 2709–2714. 10.3892/ol.2014.2553 25364454 PMC4214501

[mgg31818-bib-0022] Papadaki, I. , Mylona, E. , Giannopoulou, I. , Markaki, S. , Keramopoulos, A. , & Nakopoulou, L. (2005). PPARgamma expression in breast cancer: Clinical value and correlation with ERbeta. Histopathology, 46(1), 37–42. 10.1111/j.1365-2559.2005.02056.x 15656884

[mgg31818-bib-0023] Patel, L. , Pass, I. , Coxon, P. , Downes, C. P. , Smith, S. A. , & Macphee, C. H. (2001). Tumor suppressor and anti‐inflammatory actions of PPARγ agonists are mediated via upregulation of PTEN. Current Biology, 11(10), 764–768. 10.1016/S0960-9822(01)00225-1 11378386

[mgg31818-bib-0024] Prost, S. , Relouzat, F. , Spentchian, M. , Ouzegdouh, Y. , Saliba, J. , Massonnet, G. , Beressi, J.‐P. , Verhoeyen, E. , Raggueneau, V. , Maneglier, B. , Castaigne, S. , Chomienne, C. , Chrétien, S. , Rousselot, P. , & Leboulch, P. (2015). Erosion of the chronic myeloid leukaemia stem cell pool by PPARγ agonists. Nature, 525(7569), 380–383. 10.1038/nature15248 26331539

[mgg31818-bib-0025] Reddy, R. C. , Srirangam, A. , Reddy, K. , Chen, J. , Gangireddy, S. , Kalemkerian, G. P. , Standiford, T. J. , & Keshamouni, V. G. (2008). Chemotherapeutic drugs induce PPAR‐γ expression and show sequence‐specific synergy with PPAR‐γ ligands in inhibition of non‐small cell lung cancer. Neoplasia (New York, NY), 10(6), 597. 10.1593/neo.08134 PMC2386544 18516296

[mgg31818-bib-0026] Robbins, G. T. , & Nie, D. (2012). PPAR gamma, bioactive lipids, and cancer progression. Frontiers in Bioscience (Landmark Ed), 17, 1816–1834. 10.2741/4021 PMC3409468 22201838

[mgg31818-bib-0027] Rousselot, P. , Prost, S. , Guilhot, J. , Roy, L. , Etienne, G. , Legros, L. , Charbonnier, A. , Coiteux, V. , Cony‐Makhoul, P. , Huguet, F. , Cayssials, E. , Cayuela, J.‐M. , Relouzat, F. , Delord, M. , Bruzzoni‐Giovanelli, H. , Morisset, L. , Mahon, F.‐X. , Guilhot, F. , & Leboulch, P. (2017). Pioglitazone together with imatinib in chronic myeloid leukemia: A proof of concept study. Cancer, 123(10), 1791–1799. 10.1002/cncr.30490 28026860 PMC5434901

[mgg31818-bib-0028] Safaroghli‐Azar, A. , Bashash, D. , Kazemi, A. , Pourbagheri‐Sigaroodi, A. , & Momeny, M. (2019). Anticancer effect of pan‐PI3K inhibitor on multiple myeloma cells: Shedding new light on the mechanisms involved in BKM120 resistance. European Journal of Pharmacology, 842, 89–98. 30401630 10.1016/j.ejphar.2018.10.036

[mgg31818-bib-0029] Safe, S. , Jin, U.‐H. , Hedrick, E. , Reeder, A. , & Lee, S.‐O. (2014). Minireview: Role of orphan nuclear receptors in cancer and potential as drug targets. Molecular Endocrinology, 28(2), 157–172. 10.1210/me.2013-1291 24295738 PMC3896638

[mgg31818-bib-0030] Saiki, M. , Hatta, Y. , Yamazaki, T. , Itoh, T. , Enomoto, Y. , Takeuchi, J. , Sawada, U. , Aizawa, S. , & Horie, T. (2006). Pioglitazone inhibits the growth of human leukemia cell lines and primary leukemia cells while sparing normal hematopoietic stem cells. International Journal of Oncology, 29(2), 437–443. 10.3892/ijo.29.2.437 16820887

[mgg31818-bib-0031] Sato, H. , Ishihara, S. , Kawashima, K. , Moriyama, N. , Suetsugu, H. , Kazumori, H. , Okuyama, T. , Rumi, M. A. K. , Fukuda, R. , Nagasue, N. , & Kinoshita, Y. (2000). Expression of peroxisome proliferator‐activated receptor (PPAR) γ in gastric cancer and inhibitory effects of PPARγ agonists. British Journal of Cancer, 83(10), 1394–1400. 10.1054/bjoc.2000.1457 11044367 PMC2408786

[mgg31818-bib-0032] Sekeres, M. A. , & Stone, R. M. (2002). The challenge of acute myeloid leukemia in older patients. Current Opinion in Oncology, 14(1), 24–30. 10.1097/00001622-200201000-00005 11790976

[mgg31818-bib-0033] Shen, B. , Chu, E. S. H. , Zhao, G. , Man, K. , Wu, C.‐W. , Cheng, J. T. Y. , Li, G. , Nie, Y. , Lo, C. M. , Teoh, N. , Farrell, G. C. , Sung, J. J. Y. , & Yu, J. (2012). PPARgamma inhibits hepatocellular carcinoma metastases in vitro and in mice. British Journal of Cancer, 106(9), 1486–1494. 10.1038/bjc.2012.130 22472882 PMC3341869

[mgg31818-bib-0034] Sporn, M. B. , Suh, N. , & Mangelsdorf, D. J. (2001). Prospects for prevention and treatment of cancer with selective PPARγ modulators (SPARMs). Trends in Molecular Medicine, 7(9), 395–400. 10.1016/S1471-4914(01)02100-1 11530334

[mgg31818-bib-0035] Sun, W.‐H. , Chen, G.‐S. , Ou, X.‐L. , Yang, Y. E. , Luo, C. , Zhang, Y. , Shao, Y. , Xu, H.‐C. , Xiao, B. , Xue, Y.‐P. , Zhou, S.‐M. , Zhao, Q.‐S. , & Ding, G.‐X. (2009). Inhibition of COX‐2 and activation of peroxisome proliferator‐activated receptor γ synergistically inhibits proliferation and induces apoptosis of human pancreatic carcinoma cells. Cancer Letters, 275(2), 247–255. 10.1016/j.canlet.2008.10.023 19056168

[mgg31818-bib-0036] Suzuki, S. , Mori, Y. , Nagano, A. , Naiki‐Ito, A. , Kato, H. , Nagayasu, Y. , Kobayashi, M. , Kuno, T. , & Takahashi, S. (2016). Pioglitazone, a peroxisome proliferator‐activated receptor γ agonist, suppresses rat prostate carcinogenesis. International Journal of Molecular Sciences, 17(12), 2071. 10.3390/ijms17122071 PMC5187871 27973395

[mgg31818-bib-0037] Teresi, R. E. , Shaiu, C.‐W. , Chen, C.‐S. , Chatterjee, V. K. , Waite, K. A. , & Eng, C. (2006). Increased PTEN expression due to transcriptional activation of PPARγ by Lovastatin and Rosiglitazone. International Journal of Cancer, 118(10), 2390–2398. 10.1002/ijc.21799 16425225

[mgg31818-bib-0038] To, K. K. , Wu, W. K. , & Loong, H. H. (2018). PPARgamma agonists sensitize PTEN‐deficient resistant lung cancer cells to EGFR tyrosine kinase inhibitors by inducing autophagy. European Journal of Pharmacology, 823, 19–26. 29378193 10.1016/j.ejphar.2018.01.036

[mgg31818-bib-0039] Tsubaki, M. , Takeda, T. , Tomonari, Y. , Kawashima, K. , Itoh, T. , Imano, M. , Satou, T. , & Nishida, S. (2018). Pioglitazone inhibits cancer cell growth through STAT3 inhibition and enhanced AIF expression via a PPARγ‐independent pathway. Journal of Cellular Physiology, 233(4), 3638–3647. 10.1002/jcp.26225 29030979

[mgg31818-bib-0040] Zhang, Y. , Ba, Y. I. , Liu, C. , Sun, G. , Ding, L. I. , Gao, S. , Hao, J. , Yu, Z. , Zhang, J. , Zen, K. E. , Tong, Z. , Xiang, Y. , & Zhang, C.‐Y. (2007). PGC‐1α induces apoptosis in human epithelial ovarian cancer cells through a PPARγ‐dependent pathway. Cell Research, 17(4), 363–373. 10.1038/cr.2007.11 17372612

